# Pro-inflammatory effects of a litchi protein extract in murine RAW264.7 macrophages

**DOI:** 10.1038/hortres.2016.17

**Published:** 2016-05-04

**Authors:** Xiaoli Wang, Xiaorong Hu, Huiqing Yan, Zhaocheng Ma, Xiuxin Deng

**Affiliations:** 1 Key Laboratory of Horticultural Plant Biology, College of Horticulture and Forestry Sciences, Huazhong Agricultural University, Wuhan 430070, China; 2 Department of Cardiology, Renmin Hospital of Wuhan University, Wuhan 430070, China

## Abstract

It has been observed that the consumption of litchi often causes symptoms characterized by itching or sore throat, gum swelling, oral cavity ulcers and even fever and inflammation, which significantly impair the quality of life of a large population. Using the RAW264.7 cell line, a step-by-step strategy was used to screen for the components in litchi fruits that elicited adverse reactions. The adverse reaction fractions were identified by mass spectrometry and analyzed using the SMART program, and a sequence alignment of the homologous proteins was performed. MTT tests were used to determine the cytotoxicity of a litchi protein extract in RAW264.7 macrophages, and real-time PCR was applied to analyze the expression of inflammatory genes in the RAW264.7 cells treated with lipopolysaccharide or the litchi protein extract. The results showed that the litchi water-soluble protein extract could increase the production of the pro-inflammatory mediators *IL-1β*, *iNOS* and *COX-2*, and the anti-inflammatory mediator *HO-1* in the RAW264.7 cell line. The 14-3-3-like proteins GF14 lambda, GF14 omega and GF14 upsilon were likely the candidate proteins that caused the adverse effects.

## Introduction

Adverse food reactions are a worldwide problem, with evidence of increasing prevalence in many countries.^1^ Adverse food reactions constitute a broad term representing any abnormal clinical response associated with the ingestion of a food. In China, a large population of people suffers from shang huo, which significantly impairs their quality of life. Shang huo (rising heat) is a popular medical concept originating in traditional Chinese medicine, and it is very common in Asia.^2^ For 2000 years, Chinese and Indian people have believed that certain foods are either ‘heating’ or ‘cooling’ in the body when eaten.^3^ Litchi, longan, mandarin orange, civet durian and peanut, among others, are considered ‘heating’ fruits, and over-consumption of these fruits (dose-dependent, some people only eat one fruit) can cause shang huo.

Shang huo is likely to be a multifactorial condition involving a number of different mechanisms, although the prominence of any particular factor may vary from patient to patient. The symptoms of shang huo include the following: a burning sensation in the lips, mouth and eyes; itching and swelling of the lips, throat and gums; flushing; constipation; diarrhea; a rough yellow tongue; fever; and inflammation.

Research into shang huo has been hampered by a lack of standardization in terms of its definition and severity. The RAW264.7 murine macrophage cell line, which is frequently used as an *in vitro* model in studies of inflammation,^4^ releases a number of pro-inflammatory mediators, such as *IL-1β, iNOS* and *COX-2*, and the anti-inflammatory mediator *HO-1*, after stimulation.^5^ The bacterial endotoxin lipopolysaccharide (LPS) is frequently used as a stimulus to induce an inflammatory response.^6^ Researchers have found that components of ‘heating’ and ‘cooling’ foods may enhance or inhibit inflammatory cytokine production and potentially affect inflammation. Huang *et al.*^3^ found that water-soluble extracts from traditional ‘heating’ foods, such as litchi and longan pulps, could increase cyclooxygenase-2 (COX-2) protein levels and prostaglandin E2 production by the RAW264.7 murine macrophage cell line. In our previous study, we found that pro-inflammatory substances of the ‘heating’ fruit satsuma consisted of specific proteins by performing a systematic screen of substances from satsuma fruit in the RAW264.7 murine macrophage cell line.^7^ In contrast, LPS-induced prostaglandin E2 production was inhibited by an extract from the ‘cooling’ fruit bitter gourd. The expression levels of NF-kappa B, iNOS and COX-2 proteins were significantly inhibited by wild bitter gourd extracts.^8^

Litchi (*Litchi chinensis* Sonn.) is becoming increasingly popular among domestic and foreign consumers in recent years because it is a juicy fruit with sweet pulp and an attractive red pericarp.^9^ In addition, litchi fruits contain several nutritional and functional compounds, such as vitamin C^10^ and phenolic compounds.^11^ Adverse food reactions caused by litchi have also been reported, and the symptoms have been explained from the perspective of an allergic reaction.^12–15^ Adverse reactions to litchi occur frequently, which has severely limited its market. Previous studies have reported that some patients suffer from urticaria, erythema, pruritus, swelling of the lips, swelling of the throat, dyspnea or diarrhea after eating litchi fruits.^12–15^ As the number of clinical cases with adverse reactions to litchi increases, there is an urgent need for corresponding studies.

It has been observed that the consumption of a certain amount of litchi often causes symptoms characterized by an itchy or sore throat, swelling of the gums, oral cavity ulcer and even fever and inflammation. A step-by-step strategy was used to screen the components from litchi fruits that produce adverse effects in the RAW 264.7 macrophage line. In our study, we fractionated the edible portion of litchi using dialysis bags and protein extraction, evaluated the ability of the extract to induce the production of pro-inflammatory mediators and identified their composition by mass spectrometry using the SMART program (http://smart.embl-heidelberg.de/) and sequence alignments of homologous proteins.

## Materials and methods

### Materials and chemicals

LPS and thiazolyl blue (MTT) were purchased from Sigma-Aldrich (St. Louis, MO, USA). RPMI-1640 medium, fetal bovine serum and penicillin/streptomycin solution were from Hyclone. All molecular reagents were obtained from Toyobo (Osaka, Japan) or TAKARA (Otsu, Japan). Fruits were purchased from a local market.

### Pretreatment of the litchi samples and protein extraction

Fresh litchi fruits were peeled and pitted, and the resulting pulps were liquefied using a blender. The homogenates were filtered through four layers of gauze. Then, all filtrates were centrifuged at 5000 r.p.m. for 15 min at 4 °C. The clear supernatant solution called LWE (litchi water extract) was freeze-dried to obtain water-soluble extract samples. LWE was dissolved in water, dialyzed (cutoff point, 8 kDa) against distilled water for 5 days at 4 °C, and then freeze-dried to obtain a powder called high molecular weight LWE (HLWE). The freeze-dried samples were stored at −80 °C before protein extraction.

The HLWE powder was fully resuspended in extraction buffer (700 mM sucrose, 100 mM KCl, 500 mM Tris base, 63.7 mM EDTA, and 1 mM PMSF, pH=7.5). After adding an equal volume of Tris-phenol, the mixture was shaken for 2 h at 4 °C, centrifuged, and then the upper phenolic phase was collected. These steps were repeated twice. Afterwards, cold 0.1 M ammonium acetate in methanol was added to the phenol phase and incubated overnight at −20 °C. The collected deposits were washed three times. The protein extract was dried under nitrogen at 4 °C and called litchi water-soluble protein extract (LWP); its protein contents were quantified using a bicinchoninic acid (BCA) Protein Assay kit (Thermo Scientific, San Jose, CA, USA) according to the manufacturer’s protocol. Then, the LWP sample was analyzed by sodium dodecyl sulfate–polyacrylamide gel electrophoresis (SDS–PAGE) , and the LWP powder was stored at −80 °C until use.

### Cell culture

The RAW264.7 murine macrophage cells were grown in 25 cm^2^ flasks in RPMI-1640 medium containing 10% fetal bovine serum with penicillin (100 U mL^−1^) and streptomycin (100 μg mL^−1^). The cells were incubated at 37 °C and 5% CO_2_ in a fully humidified incubator. When the cells reached confluence, they were harvested with a cell scraper and diluted with fresh complete medium.

### Cytotoxicity test

A cytotoxicity test was performed using the MTT method. The RAW264.7 cells were removed using a cell scraper and were seeded into 96-well plates (100 μL per well) at a density of 5×10^3^ cells per well. After a 24-h incubation at 37 °C and 5% CO_2_, the cells were treated with serum-free RPMI-1640 containing various concentrations of LWP (0–1 mg mL^−1^) and cultured for an additional 12 h. Then, 20 μL of the MTT solution (5 mg mL^−1^ in phosphate-buffered saline buffer solution) was added to each well, and the cells were incubated continuously. Approximately 4 h later, the medium was removed and the resulting formazan product was dissolved with 100 μL of dimethyl sulfoxide. Finally, the optical density was measured at 490 nm^16^ using a microplate reader. All assays were performed in quintuplicate. The results are presented as a percentage of the control.

### Semi-quantitative PCR and real-time reverse transcription PCR analysis

The cells were seeded into 24-well plates. After 24 h, when the cells had adhered to the bottom of the well, the medium was replaced with serum-free medium containing 0.5 mg mL^−1^ LWP or 10 μg mL^−1^ LPS. The cells were incubated for 12 h under the same conditions and then collected for the reverse transcription PCR analysis.

The total RNA was extracted from the RAW 264.7 cells using Trizol reagent, according to the manufacturer’s protocol. The extracted RNA was reverse transcribed using an RNA PCR Kit and an oligo adapter primer.^17^ The *β-actin* gene, a constitutively expressed gene, was analyzed as an internal standard, and other genes were amplified. The primers for each gene are as follows: *β-actin* Forward primer: 5′-TGAAGGGCATCTTGGGCTACAC-3′ *β-actin* Reverse primer: 5′-TGGGTGGTCCAGGGTTTCTTAC-3′; *COX-2* Forward primer: 5′-ATCTGGCTTCGGGAGCACAAC-3′ *COX-2* Reverse primer: 5′-GAGGCAATGCGGTTCTGATACTG-3′; *iNOS* Forward primer: 5′-GTCTTGGTGAAAGTGGTGTT-3′ *iNOS* Reverse primer: 5′-GTGCTTGCCTTATACTGGTC-3′; *HO-1* Forward primer: 5′-CGGGCCAGCAACAAAGTG-3′ *HO-1* Reverse primer: 5′-AGTGTAAGGACCCATCGGAGA-3′; *IL-1β* Forward primer: 5′-GTTGACGGACCCCAAAAGAT-3′ *IL-1β* Reverse primer: 5′-CCTCATCCTGGAAGGTCCAC-3.

The reverse transcription PCR mixture consisted of 1 μL of complementary DNA, 10.5 μL of ddH_2_O (RNase-free water), 12.5 μL of mix, 0.5 μL of forward primer and 0.5 μL of reverse primer. Amplification was performed for 30 cycles: denaturation at 94 °C for 30 s, annealing at 55 °C for 1 min and extension at 72 °C for 30 s. The fragments were separated on 1% (w/v) agarose gels by electrophoresis.

Quantitative real-time PCR was performed in a LightCycler instrument (Roche, Mannheim, Germany) with the FastStart DNA Master SYBR Green I Kit. The following components were added to the PCR mixture (10 μL): 0.5 μL of complementary DNA, 3.5 μL of ddH_2_O (RNase-free water), 5 μL of SYBR Green I qPCR Mix (Toyobo), 0.5 μL of forward primer and 0.5 μL of reverse primer. The reactions were cycled as follows: 50 °C for 2 min, 95 °C for 10 min; 95 °C for 15 s, 60 °C for 1 min (40 cycles). Relative gene expression was calculated using the 2^−ΔΔCt^ method.^18^

### Mass spectrometry

The proteins were identified at APT (Shanghai Applied Protein Technology Co., Ltd., shanghai, China) using a shotgun proteomic method. The proteins were digested using the filter-aided sample preparation (FASP) procedure described by Wisniewski *et al.*^19^ In brief, the protein pellet was dissolved in SDT buffer (4% SDS, 100 mM DTT, 150 mM Tris-HCl pH 8.0) at 90 °C for 5 min, followed by several ultrafiltration steps (microcon units, 30 kD) and blocking with 0.05 M iodoacetamide UA buffer (8 M urea and 150 mM Tris-HCl, pH 8.0) in the dark for 20 min. Finally, the protein suspension was digested with trypsin (Promega, Madison, WI, USA) in 25 mM NH_4_HCO_3_ overnight at 37 °C, and the resulting peptides were contained in the resulting filtrate. The tryptic peptide mixtures were desalted and separated using an Ettan multidimensional liquid chromatography (MDLC) system (GE Healthcare, Piscataway, NJ, USA). Mobile phase A was 0.1% formic acid in high-performance liquid chromatography-grade water and mobile phase B was 0.1% formic acid in acetonitrile. The samples were loaded onto reversed-phase (RP) trap columns (Zorbax 300 SB C18, Agilent Technologies, Wilmington, DE, USA) and separated at a flow rate of 2 μL min^−1^ using the following linear gradient: 4–50% buffer B for 50 min, 50–100% buffer B for 4 min and 100% buffer B for 6 min. Data-dependent MS/MS (tandem mass spectrometry) spectra were acquired by detecting peptides with an light-triggered and light-quenched (LTQ) Velos (Thermo Scientific, San Jose, CA, USA). Each scan cycle consisted of one full scan mass spectrum (*m*/z 300–1800), followed by 20 MS/MS events of the most intense ions.

### Statistical analysis

The data are presented as the mean±s.d. Statistical significance was determined with one-way analysis of variance (ANOVA) followed by Dunnett’s test. *P*-values <0.05 were considered statistically significant.

## Results

### Extraction and SDS–PAGE analysis of LWP from litchi fruits

**Figure 1:**
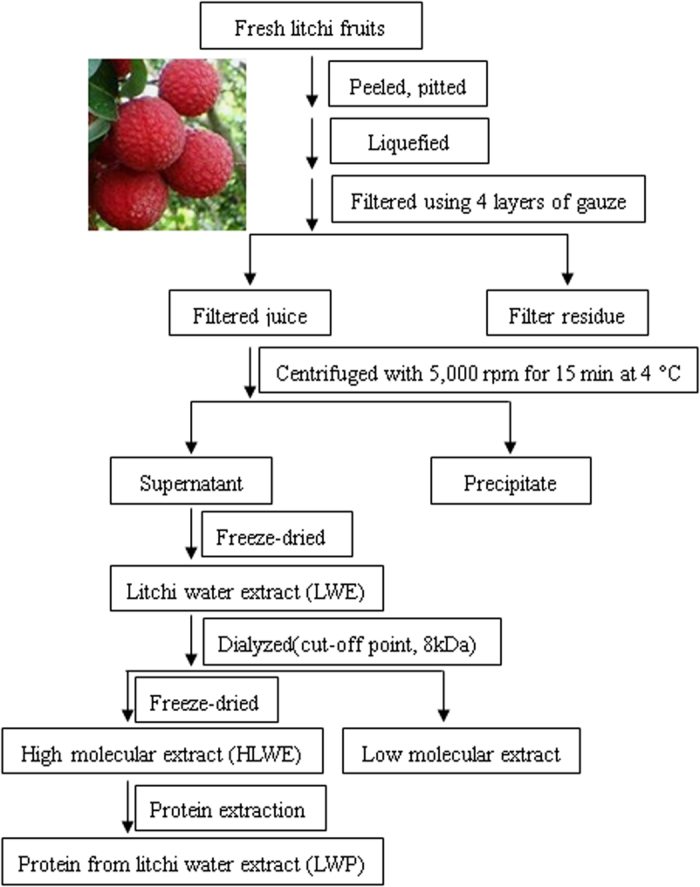
The extraction methods used to determine the composition of the pro-inflammatory compounds (litchi water-soluble protein extract (LWP)) in litchi fruits. High-molecular-weight LWE (HLWE) and LWP were stored at −80 °C until use.

**Figure 2:**
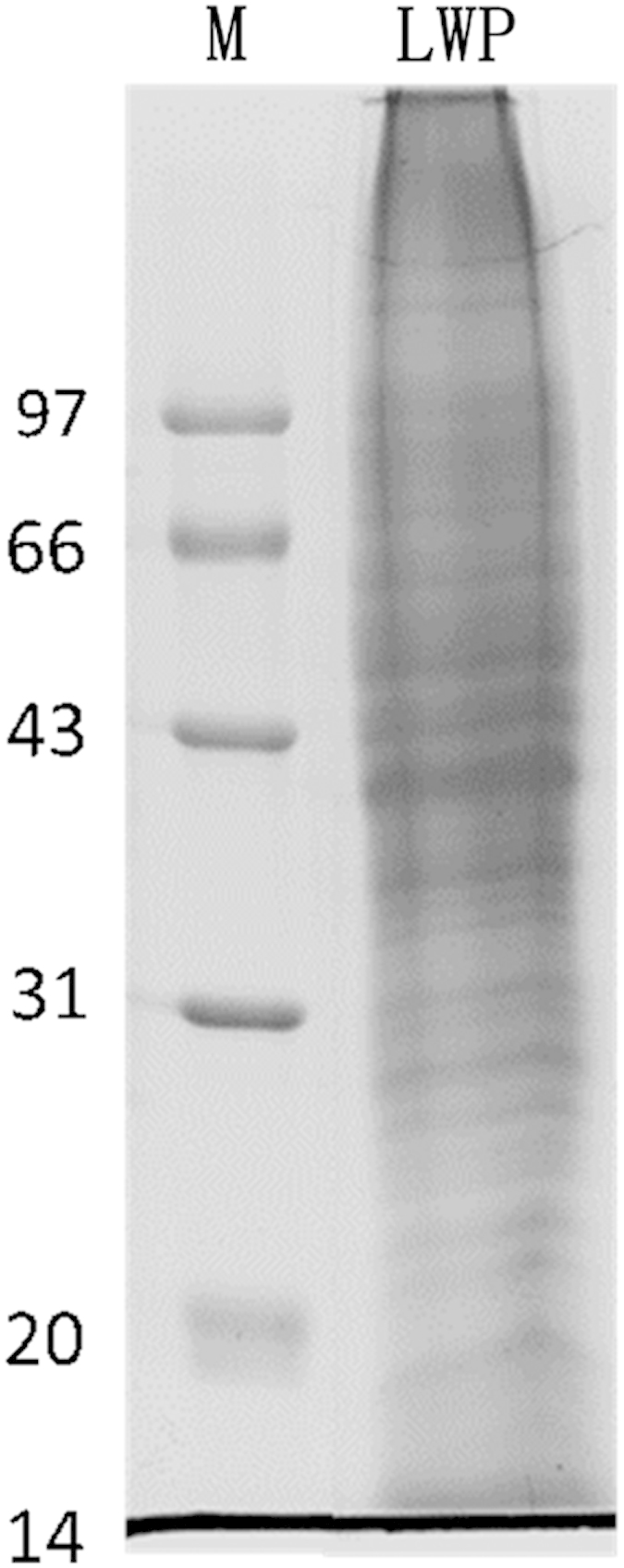
The litchi water-soluble protein extract (LWP) extracted from litchi was analyzed by sodium dodecyl sulfate–polyacrylamide gel electrophoresis (SDS–PAGE). The molecular marker is in the left lane and LWP is in the right lane.

A step-by-step strategy was used to isolate the pro-inflammatory compounds from litchi fruit, which were analyzed in the RAW 264.7 macrophage cell line, as shown in Figure 1. The LWE was separated using dialysis into a HLWE and a low-molecular-weight fraction. The LWP proteins were extracted from the HLWE fraction. The LWP extract was separated by SDS–PAGE. As shown in Figure 2, there were several protein bands in the PAGE gel, indicating that the LWP contained many proteins. The pro-inflammatory effects of the fractions were tested in the RAW 264.7 cell line.

### Identification of LWP proteins

**Figure 3:**
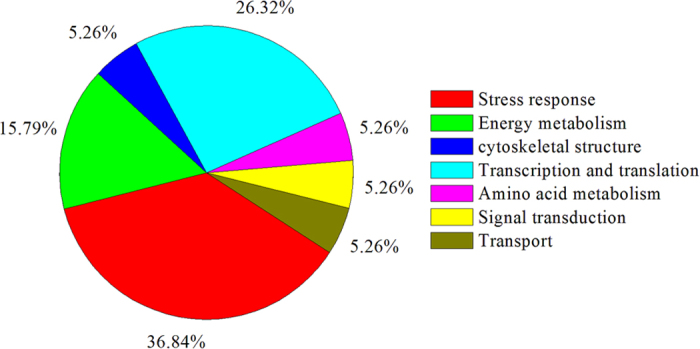
Functional categories of the identified proteins.

The LWP proteins in the extract were identified by liquid chromatography-MS/MS. The MS/MS spectra were automatically searched against the UniProt protein database (http://www.uniprot.org/; downloaded May 2014) using the Bioworks Browser rev. 3.1 (Thermo Electron, San Jose, CA, USA). The results were obtained from SEQUEST out files with a Build Summary and were filtered based on the following criteria: charge=1, Xcorr⩾1.9; charge=2, Xcorr⩾2.2; charge=3, Xcorr⩾3.75; and Delta CN⩾0.1. A high level of confidence was obtained for 19 proteins (number of unique peptides ⩾2), shown in Table 1. These proteins can be classified into seven groups according to their functions. Seven stress-response proteins: glyceraldehyde-3-phosphate dehydrogenase GAPC1, probable mediator of RNA polymerase II transcription subunit 37c, 5-methyltetrahydropteroyltriglutamate–homocysteine methyltransferase 1, 14-3-3-like protein GF14 lambda, 14-3-3-like protein GF14 omega, 14-3-3-like protein GF14 upsilon and Ras-related protein RABG3e. Three energy metabolism-related proteins: ATP synthase subunit alpha, ATP synthase subunit beta-3 and ATPase 10. 1 cellular structure protein: actin-7. 5 transcription- and translation-related proteins: mediator of RNA polymerase II transcription subunit 37a, ubiquitin-40S ribosomal protein S27a-1, elongation factor tu, elongation factor 1-alpha 1 and eukaryotic initiation factor 4A-1. 1 amino acid metabolism-related protein: adenosylhomocysteinase 2. 1 signal transduction-related protein: calmodulin-1. 1 transport protein: ADP-ribosylation factor 1. The percentages of proteins represented in each of the seven groups are shown in Figure 3.

### Effects of LWP on cell viability

**Figure 4:**
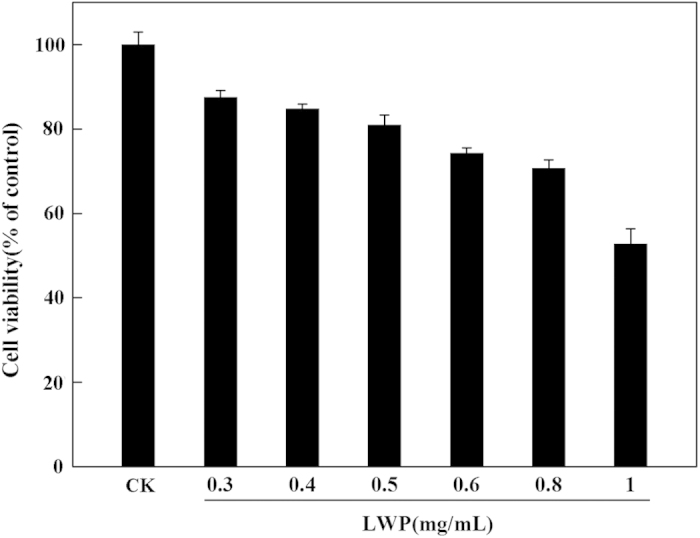
The viability of RAW264.7 murine macrophage cells was measured using MTT assays. The cells were treated with serum-free RPMI-1640 containing various concentrations of LWP (0.3–1 mg mL^−1^) for 12 h. The results are presented as a percentage of the control (CK: untreated cells). The data shown are the mean±s.d. of five determinations.

The cytotoxicity of the LWP was tested in RAW264.7 cells using the MTT assay. The cells were treated with various concentrations of LWP (0–1 mg mL^−1^) and LPS (10 μg mL^−1^). When the concentration of LWP was <0.5 mg mL^−1^, the cells exhibited good viability (>80.95%) and a high density. The cell viability was between 74.25 and 72.19% after the cells were treated with 0.6–0.8 mg mL^−1^ LWP. Then, as the concentration of LWP was increased to ~1 mg ml^−1^, the cell viability was reduced to 52.76% (Figure 4). The cell viability was 80.55% after the cells were treated with 10 μg mL^−1^ LPS, which was similar to treatment with 0.5 mg mL^−1^ LWP.

### Litchi proteins increased the mRNA expression levels of inflammatory mediators

**Figure 5:**
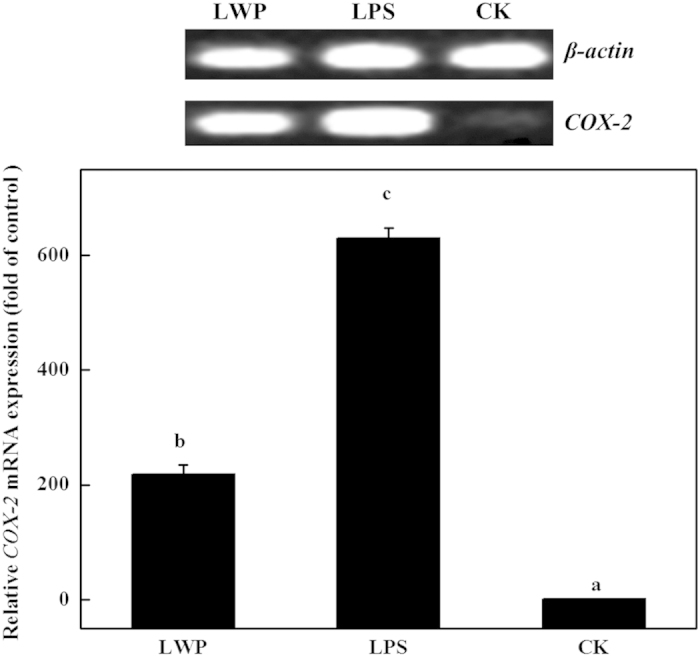
The effects of litchi water-soluble protein extract (LWP) on *COX-2* expression levels. The cells were incubated with LWP (0.5 mg mL^−1^) and lipopolysaccharide (LPS; 10 μg mL^−1^) for 12 h. The results are presented as the relative gene expression (fold change relative to the control-CK: untreated cells). The data shown are the means±s.d. of three determinations. *P*<0.05.

**Figure 6:**
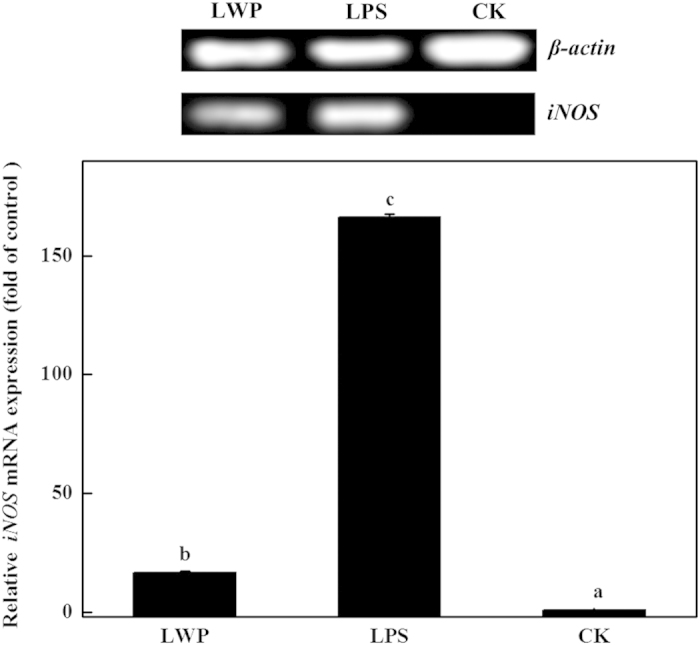
The effects of litchi water-soluble protein extract (LWP) on *iNOS* expression levels. The cells were incubated with LWP (0.5 mg mL^−1^) and lipopolysaccharide (LPS; 10 μg mL^−1^) for 12 h. The results are presented as the relative gene expression (fold change relative to the control-CK: untreated cells). The data shown are the mean±s.d. of three determinations. *P*<0.05.

**Figure 7:**
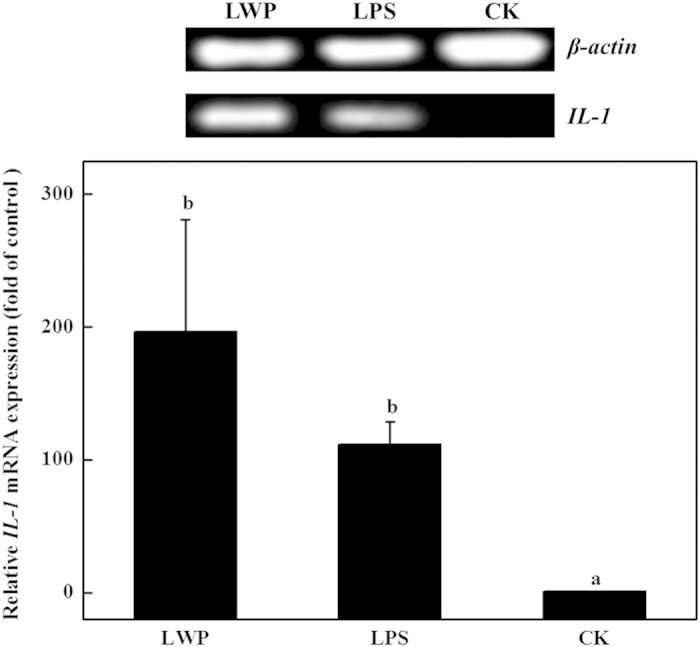
The effects of litchi water-soluble protein extract (LWP) on *IL-1* expression levels. The cells were incubated with LWP (0.5 mg mL^−1^) and lipopolysaccharide (LPS; 10 μg mL^−1^) for 12 h. The results are presented as the relative gene expression (fold change relative to the control-CK: untreated cells). The data shown are the mean±s.d. of three determinations. *P*<0.05.

**Figure 8:**
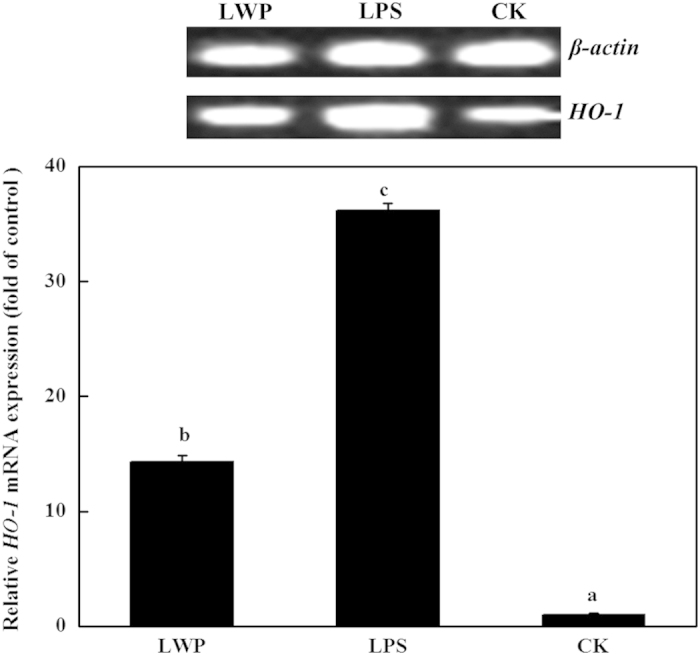
The effects of litchi water-soluble protein extract (LWP) on *HO-1* expression levels. The cells were incubated with LWP (0.5 mg mL^−1^) and lipopolysaccharide (LPS; 10 μg mL^−1^) for 12 h. The results are presented as the relative gene expression (fold change relative to the control-CK: untreated cells). The data shown are the mean±s.d. of three determinations. *P*<0.05.

The effects of LWP on *COX-2*, *iNOS*, *HO-1* and *IL-1β* expression were analyzed by reverse transcription PCR and real-time PCR. LPS was used as the positive control, and serum-free RPMI-1640 medium was used as the negative control. As shown in Figure 5, the LPS-treated and LWP-induced groups showed distinct increases in *COX-2* expression levels, and the values were ~630-fold and 218-fold higher than the control, respectively. The expression of *iNOS* exhibited a similar increasing trend as *COX-2* in the three groups (Figure 6). The *iNOS* expression levels in the LPS-treated RAW 264.7 cells were ~166-fold higher than that in the control and were 17-fold higher in the LWP-induced group. The LPS and LWP treatments also increased cellular *IL-1β* levels, but *IL-1β* expression levels in the LPS-stimulated cells (111-fold) were lower than in the LWP-treated cells (196-fold; Figure 7). These changes indicated that LWP should play an important role in the pro-inflammatory effects of litchi, and its pro-inflammatory mechanism may be distinguished from conventional LPS. Moreover, LPS (36-fold) and LWP (14-fold) notably stimulated the release of the anti-inflammatory mediator *HO-1* (Figure 8). Therefore, pro-inflammatory and anti-inflammatory responses exist in the immune activation of the LPS- or LWP-treated RAW 264.7 cells.

### Functional analysis of the proteins

The mass spectra of the proteins were further analyzed and processed. The conserved domains were predicted by the SMART program. Homologous proteins in *Homo sapiens* and *Mus musculus* were identified, and a sequence alignment with homologous proteins was performed. The results reveal that the 14-3-3-like proteins GF14 lambda, GF14 omega and GF14 upsilon were likely the candidate pro-inflammatory proteins rather than the other detected proteins. These proteins belong to the 14-3-3 family and have a conserved 14-3-3 domain (Table 2).

## Discussion

It is well known that eating horticultural fruits is healthy and can prevent some diseases. However, the adverse reactions caused by horticultural fruits should receive more attention.^20,21^ In China, shang huo disease induced by the intake of ‘heating’ fruits occurs at a high frequency, and the process of shang huo is always accompanied by inflammation. Litchi, a typical ‘heating’ fruit, was studied to screen for pro-inflammatory components and their mechanisms of action in this study.

Based on previous studies by Yan *et al.*^7^ and Huang *et al.*,^3^ we found that the pro-inflammatory components of litchi were present in water-soluble extracts and were likely to be proteins. The LWP extract from litchi fruit significantly activated the release of the pro-inflammatory mediators *IL-1β*, *iNOS* and *COX-2*, and the anti-inflammatory mediator *HO-1* in the RAW264.7 murine macrophage cell line. This result was similar to LPS, which can stimulate a strong immune response in animals.^22^ An abundant release of these inflammatory mediators could indicate that both pro-inflammatory and anti-inflammatory responses exist in the same immune course. It was interesting to note that the expression levels of *iNOS*, *COX-2* and *HO-1* in the LWP-treated cells were lower than those in the LPS-induced group, whereas the expression levels of *IL-1β* were higher in the LWP-treated cells. Therefore, LWP should play an important role in the pro-inflammatory effects of litchi, and its pro-inflammatory mechanism may be distinguished from conventional LPS. This mechanism requires further study.

**Figure 9:**
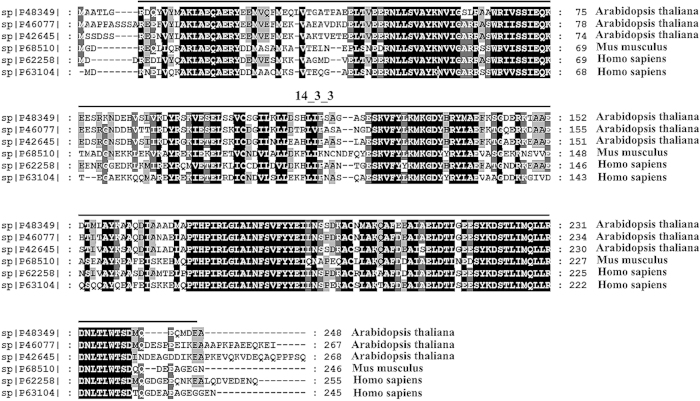
Alignment of the amino acid sequences of some homologous proteins from the 14-3-3 family. The black and gray shadings indicate sequence identity and similarity, respectively. The conserved motifs in 14-3-3 are indicated by the lines above the sequence. The accession nos. from UniProt were provided on the left of the sequence and the species names are on the right.

By analyzing the mass spectrometry results, we identified members of the 14-3-3 family that are associated with resistance to fungal and bacterial pathogens in many plants.^23–25^ The components of the plant defense system themselves may induce adverse reactions in the body.^7^ In addition, the multiple sequence alignment showed that some 14-3-3 proteins from *Arabidopsis thaliana*, *Mus musculus* and *Homo sapiens* are highly homologous (Figure 9). Previous reports have shown a close relationship between 14-3-3 proteins and inflammation or cancers.^26–31^ The 14-3-3-like proteins GF14 lambda, GF14 omega and GF14 upsilon were considered the most likely candidate pro-inflammatory proteins, rather than other detected proteins. These proteins belong to the 14-3-3 family and have a conserved 14-3-3 domain, as predicted by SMART (Table 2). The multiple sequence alignment showed that some 14-3-3 proteins from *Arabidopsis thaliana*, *Mus musculus* and *Homo sapiens* are highly homologous (Figure 9). Members of the 14-3-3 family play important roles in a number of biological processes, such as kinase-mediated signal transduction, the regulation of growth and development, and the response to environmental stress.^32^ Meanwhile, reports have shown that levels of the 14-3-3η and 14-3-3γ proteins were significantly increased in the synovial fluid and serum samples from patients with inflammation-related diseases,^26^ and the pathway by which MAPKAPK2-mediated phosphorylation regulates 14-3-3ζ functions may be related to p38 MAPK-dependent inflammation.^27^ Many 14-3-3 proteins have been shown to regulate tumor progression. 14-3-3ε overexpression contributes to non-small cell lung cancer progression^28^ and hepatocellular carcinoma cell migration.^29^ 14-3-3ε serves as a target for the prevention and therapy of colorectal cancer. Nonsteroidal anti-inflammatory drugs exert their actions by inhibiting 14-3-3ε in treatments for colorectal cancer.^30^ However, 14-3-3σ can suppress the neoplastic transition of breast epithelial cells.^31^ Inflammatory responses have been linked to cancer initiation and progression,^33^ which can promote the release of inflammatory mediators in *in vivo* and *in vitro* models. Our results demonstrate that the 14-3-3-like proteins GF14 lambda, GF14 omega and GF14 upsilon from litchi may induce the production of inflammatory mediators.

Generally, the activation of inflammation can induce the production of a large number of pro-inflammatory cytokines, which subsequently stimulate macrophages to release anti-inflammatory mediators that inhibit the expression of pro-inflammatory cytokine genes and suppress the inflammatory response.^34–36^ A well-adapted anti-inflammatory response during the progression of inflammation may prevent it from evolving into systemic inflammation^37^ or cancer. The consumption of large amounts of ‘heating’ foods, such as litchi, longan, peach, satsuma and others, can induce shang huo diseases, which induce the expression of a wide range of inflammatory mediators. However, the less severe shang huo symptoms usually disappear as a result of autoimmunity after a period of time, which may strengthen the functioning of the immune system to some extent by increasing the levels of anti-inflammatory mediators in the body. In addition, the degree of shang huo disease varies from person to person, which may be associated with the degree of protein degradation and the functions of the immune system, such as the production of anti-inflammatory mediators. In summary, the present *in vitro* study (RAW264.7 murine macrophage cell line) supports the hypothesis that shang huo disease is closely related to inflammation. The LWP extract from litchi can induce the expression of a wide range of inflammatory mediators, and the 14-3-3-like proteins GF14 lambda, GF14 omega and GF14 upsilon were considered the proteins that most likely induced shang huo symptoms according to a mass spectrometric analysis of LWP.

### Conclusion

In conclusion, a novel extract named LWP has been isolated from litchi fruits. The LWP fraction can increase the expression levels of the pro-inflammatory mediators *IL-1β*, *iNOS* and *COX-2* and the anti-inflammatory mediator *HO-1* by the RAW264.7 murine macrophage cell line. Among the identified proteins, the 14-3-3-like proteins GF14 lambda, GF14 omega and GF14 upsilon were considered the most likely candidate pro-inflammatory proteins, rather than the other detected proteins, according to the functional analysis by the SMART program. They share high amino acid sequence similarity with certain homologous proteins in *Homo sapiens* and *Mus musculus*. All of the results showed that these proteins may cause shang huo symptoms, and shang huo diseases may be closely related to inflammatory responses.

## Figures and Tables

**Table 1:** Potential proteins identified by liquid chromatography-MS/MS

*Accession no.*	*Protein name*	*MW (molecular weight)*
sp|P53492	Actin-7	41735.83
sp|P36397	ADP-ribosylation factor 1	20608.6
sp|P25858	Glyceraldehyde-3-phosphate dehydrogenase GAPC1, cytosolic	36914.16
sp|Q9LHA8	Probable mediator of RNA polymerase II transcription subunit 37c	71101.38
sp|O50008	5-methyltetrahydropteroyltriglutamate–homocysteine methyltransferase 1	84356.66
sp|Q9LKR3	Mediator of RNA polymerase II transcription subunit 37a	73629.23
sp|P48349	14-3-3-like protein GF14 lambda	27975.74
sp|P59271	Ubiquitin-40S ribosomal protein S27a-1	17671.5
sp|P17745	Elongation factor Tu, chloroplastic	51630.12
sp|Q01525	14-3-3-like protein GF14 omega	29161.83
sp|P42645	14-3-3-like protein GF14 upsilon	30182.02
sp|P05492	ATP synthase subunit alpha, mitochondrial	55596.9
sp|Q9C5A9	ATP synthase subunit beta-3, mitochondrial	59859.18
sp|Q43128	ATPase 10, plasma membrane-type	104815.49
sp|P0DH99	Elongation factor 1-alpha 1	49502.13
sp|Q9LK36	Adenosylhomocysteinase 2	53159.06
sp|P0DH95	Calmodulin-1	16861.74
sp|P41376	Eukaryotic initiation factor 4A-1	46704.52
sp|Q9XI98	Ras-related protein RABG3e	22979.84

**Table 2:** Data derived from UniProt and the SMART program

*Protein name*	*MW*	*Sequence similarities*	*Graphical scheme adapted from SMART*
14-3-3-like protein GF14 lambda	27975.7	14-3-3 Family	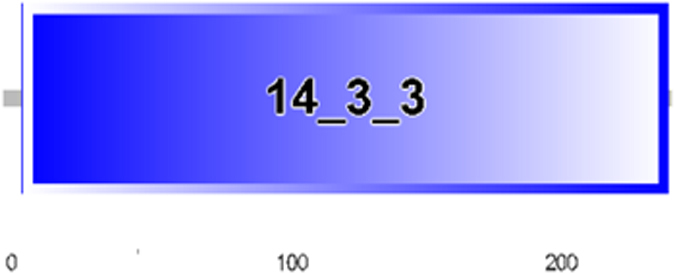
14-3-3-like protein GF14 omega	29161.8	14-3-3 Family	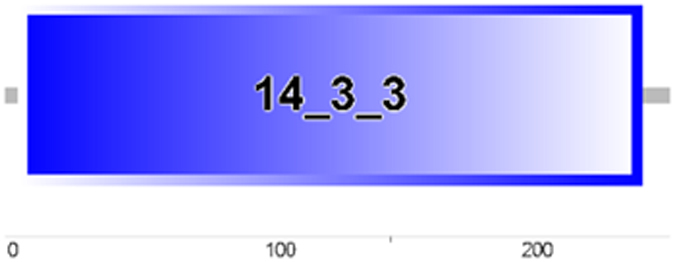
14-3-3-like protein GF14 upsilon	30182	14-3-3 Family	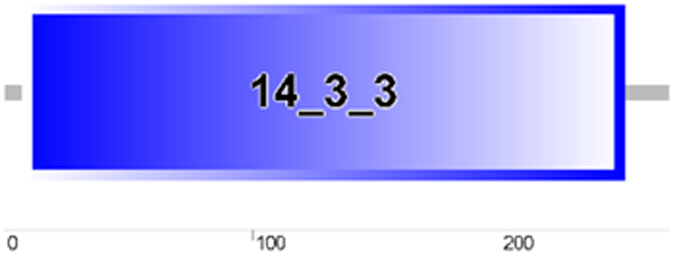

The sequence similarities and protein domains are provided.
