# Pro-inflammatory effects of a litchi protein extract in murine RAW264.7 macrophages

**DOI:** 10.1038/hortres.2017.59

**Published:** 2017-10-25

**Authors:** Xiaoli Wang, Xiaorong Hu, Huiqing Yan, Zhaocheng Ma, Xiuxin Deng

**Correction to:** *Horticulture Research* (2016) **3**, 16017; doi:10.1038/hortres.2016.17; Published online 04 May 2016

Since the publication of this article, the authors have noticed an error in Figure 6, the correct Figure 6 should be:

In addition, the author Xiuxin Deng should be removed from the author list.

The authors would like to apologize for this error.

## Figures and Tables

**Figure 6:**
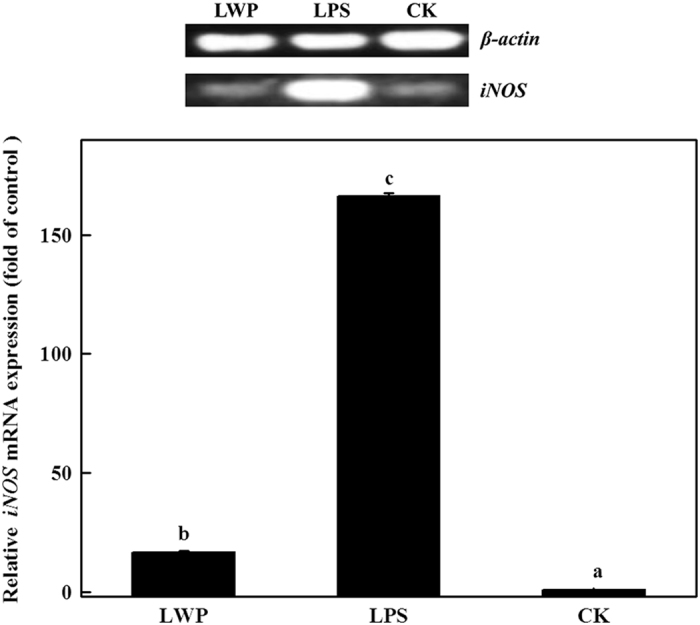
.

